# Glutamate and GABA in autism spectrum disorder—a translational magnetic resonance spectroscopy study in man and rodent models

**DOI:** 10.1038/s41398-018-0155-1

**Published:** 2018-05-25

**Authors:** Jamie Horder, Marija M. Petrinovic, Maria A. Mendez, Andreas Bruns, Toru Takumi, Will Spooren, Gareth J. Barker, Basil Künnecke, Declan G. Murphy

**Affiliations:** 1 Department of Forensic and Neurodevelopmental Sciences, Institute of Psychiatry, Psychology and Neuroscience, King’s College London, De Crespigny Park, London, SE5 8AF UK; 2 Roche Pharma Research & Early Development, Neuroscience, Roche Innovation Center Basel, F. Hoffmann-La Roche Ltd, Grenzacherstrasse 124, CH-4070 Basel, Switzerland; 3 RIKEN Brain Science Institute, Wako, Japan; 4 Centre for Neuroimaging Sciences, Institute of Psychiatry, Psychology and Neuroscience, King’s College London, De Crespigny Park, London, SE5 8AF UK; 5 Autism Assessment and Behavioural Genetics Clinic, South London and Maudsley NHS Foundation Trust, Bethlem Royal Hospital, Beckenham, UK; 6 Sackler Institute for Translational Neurodevelopment, Institute of Psychiatry, Psychology and Neuroscience, King’s College London, London, SE5 8AF United Kingdom; 7 Present Address: Department of Forensic and Neurodevelopmental Sciences, and The Sackler Institute for Translational Development, Institute of Psychiatry, Psychology and Neuroscience, King’s College London, De Crespigny Park, London, SE5 8AF UK

## Abstract

Autism spectrum disorder (ASD) is a pervasive neurodevelopmental syndrome with a high human and economic burden. The pathophysiology of ASD is largely unclear, thus hampering development of pharmacological treatments for the core symptoms of the disorder. Abnormalities in glutamate and GABA signaling have been hypothesized to underlie ASD symptoms, and may form a therapeutic target, but it is not known whether these abnormalities are recapitulated in humans with ASD, as well as in rodent models of the disorder. We used translational proton magnetic resonance spectroscopy ([1H]MRS) to compare glutamate and GABA levels in adult humans with ASD and in a panel of six diverse rodent ASD models, encompassing genetic and environmental etiologies. [1H]MRS was performed in the striatum and the medial prefrontal cortex, of the humans, mice, and rats in order to allow for direct cross-species comparisons in specific cortical and subcortical brain regions implicated in ASD. In humans with ASD, glutamate concentration was reduced in the striatum and this was correlated with the severity of social symptoms. GABA levels were not altered in either brain region. The reduction in striatal glutamate was recapitulated in mice prenatally exposed to valproate, and in mice and rats carrying *Nlgn3* mutations, but not in rodent ASD models with other etiologies. Our findings suggest that glutamate/GABA abnormalities in the corticostriatal circuitry may be a key pathological mechanism in ASD; and may be linked to alterations in the neuroligin–neurexin signaling complex.

## Introduction

Autism spectrum disorder (ASD) is a pervasive neurodevelopmental syndrome characterized by deficits in social reciprocity and communication, and by restricted interests and repetitive behaviors (DSM-5)^1^. ASD affects 1–2% of the population and is up to five times more common in males than in females^2^. The high-societal burden of ASD is exacerbated by the fact that there are no effective pharmacological treatments for the core symptoms of the disorder. This is principally because the etiology and pathophysiology of ASD are largely unclear, thus leading to a lack of therapeutic targets. Most ASD cases are idiopathic, i.e., they cannot be ascribed to any known etiological cause^3^, and while genetic and environmental risk factors accounting for some cases of ASD have been identified, the neural mechanisms involved remain uncertain.

However, emerging evidence suggests that abnormalities in the balance between excitatory (glutamate-mediated), and inhibitory (GABA-mediated) neurotransmission may be a common pathophysiological mechanism and hence a treatment target in ASD^4–6^. For instance, abnormalities in the expression of glutamate and GABA receptors have been observed in the postmortem brains of people with ASD^7^. In vivo, proton magnetic resonance spectroscopy ([1H]MRS) has revealed alterations in the levels of glutamate and glutamine in the cortex and basal ganglia of children, and in the basal ganglia in adults with ASD^8–16^. Moreover, reductions in GABA have been reported in several brain regions in children with ASD^13,17–20^. While these studies are consistent with and support the hypothesis of an excitation/inhibition (E/I) imbalance in ASD, their respective findings do not always concur in terms of the directionality of the imbalance, and the underlying biological mechanism(s) remain poorly understood^7–20^. Progress can be made, however, with the help of rodent models of ASD. These models encompass diverse etiologies including genetic, chromosomal, i.e., copy number variations (CNVs), and environmental insults^21^. This offers a powerful toolbox to explore the underpinnings of an E/I imbalance in ASD. Electrophysiological and biochemical investigations in animal models of ASD have revealed brain region- and model-specific imbalances in E/I function^6,22–26^, yet it has been difficult to relate these findings to human ASD due to the invasive nature of the techniques, e.g., brain slice recordings, that are typically used. In contrast, [1H]MRS is noninvasive and can be safely used in humans and in animals alike—thus allowing direct inter-species comparisons. Yet, comparable [1H]MRS investigations of glutamate and GABA in both humans and rodent models of ASD do not currently exist.

Therefore, in order to determine whether ASD is associated with abnormal levels of glutamate and GABA in vivo, and to explore its putative biological underpinnings, we conducted a translational [1H]MRS study in both humans with idiopathic ASD and a panel of rodent models of ASD. We selected six etiologically diverse models to cover a wide range of previously identified risk factors for non-syndromic ASD in humans, namely (1) prenatal exposure to valproate (valproic acid, VPA), an environmental risk factor for ASD^27^, (2) paternal duplication of the 15q11-13 chromosomal region, the most frequent ASD-associated CNV^28–31^, and (3) three monogenic models carrying mutations in synapse-related genes that have been linked with ASD in humans: *Shank3* knockout (KO) mice^26,32^, and *Nlgn3*^*R451C*^ knock-in (KI) mice and *Nlgn3* KO rats, both of which with mutations in the same gene, thus permitting comparisons across two rodent species^25,33^. In addition, an inbred mouse strain, the BTBR T+tf/J mice^34^, with strong face validity but unknown genetics was used as a model of ASD of presumed polygenic origin. We performed [1H]MRS in the same brain regions across humans, mice, and rats in order to allow for direct cross-species comparisons. We chose to focus on two brain areas previously implicated in both ASD pathology and social behavior in individuals without ASD, namely the striatum and the medial prefrontal cortex (mPFC)^6,11,35–37^.

## Materials and methods

### Human participants

A total of 25 individuals with idiopathic ASD were recruited from the Behavioral Genetics Clinic at the Maudsley Hospital, a national referral service for the diagnosis of neurodevelopmental disorders. Control participants were recruited via local advertisements. Inclusion criteria for all human participants included: (1) full-scale IQ of ≥80; (2) not currently taking any psychoactive medication and no psychoactive medication within at least the past six weeks; (3) no history of epilepsy, head injury, brain disease or infection, or serious medical illness (by self-report); (4) no history of bipolar disorder, schizophrenia, or drug or alcohol dependency; (5) no contraindications for MRI scanning i.e., no metallic implants, pacemakers, or history of major surgery to the head or chest. Participants in the ASD group were required to meet International Classification of Disease-Revision 10 (ICD-10) research criteria for autism. All diagnoses were confirmed using the Autism Diagnostic Observation Schedule (ADOS-G) and where possible also with the Autism Diagnostic Interview-Revised. A summary of the recruited participants’ demographics is given in Table 1. The two groups did not differ in age, gender, or in either full-scale IQ, verbal IQ or performance IQ as assessed using the Wechsler Abbreviated Scale of Intelligence. Ethical approval for this study was provided by the Essex 2 National Research Ethics Committee (reference 04-Q0102/26). Full written informed consent according to the Declaration of Helsinki was obtained from all participants.

**Table 1:** Summary of demographic and clinical characteristics of human participants

Clinical characteristics	Controls *n* = 36 (mean ± SEM)	ASD *n* = 25 (mean ± SEM)	ASD vs. control*s* *p*-value
Age (years)	28.91 ± 1.40	30.98 ± 1.81	0.36
Verbal IQ	117.29 ± 1.91	115.05 ± 3.31	0.53
Performance IQ	115.32 ± 2.26	114.32 ± 3.74	0.81
Full-scale IQ	118.66 ± 4.07	114.23 ± 3.19	0.23
ADI-R social interaction	NA	16.11 ± 1.26	NA
ADI-R communication	NA	12.58 ± 1.56	NA
ADI-R repetitive behavior	NA	4.74 ± 0.56	NA
ADOS-G social interaction	NA	7.73 ± 0.46	NA
ADOS-G communication	NA	4.00 ± 0.36	NA

The control group did not receive ADI-R or ADOS-G assessment. For reference, the widely accepted cut-offs for ASD diagnosis are: ADI-R: ≥10 (social interaction), ≥8 (communication), ≥3 (repetitive behavior); ADOS-G: ≥4 (social interaction), ≥2 (communication), ≥7 (social interaction and communication combined). Data are depicted as mean ± SEM; two-tailed *t*-test

*ADI-R* Autism Diagnostic Interview-Revised, *ADOS-G* Autism Diagnostic Observation Schedule, *ASD* autism spectrum disorder, *IQ* intelligence quotient, *NA* not applicable

### Human [1H]MRS: data acquisition and processing

The striatum (including the head of the caudate, the anterior putamen, and the internal capsule) and the mPFC were selected as regions-of-interest (ROI) on the grounds that neuropathological and MRI studies have linked these brain areas to ASD^6,11,35–37^ and that we showed a reduction in Glx (combined pools of glutamate, glutamine, and glutathione) in striatum previously^11^. As practical considerations limited us to a single unilateral striatal ROI, we chose the left striatum in order to maintain maximum compatibility with our former study^11^. Moreover, earlier studies reported independency of metabolite levels with the ROI laterality^38–40^. For the cortical ROI, the bilateral mPFC including the cingulate gyrus was selected. Supplementary Figure 1a depicts examples of ROI placements.

[1H]MRS data were acquired on a 3T GE HDxt MRI scanner (GE Medical Systems, Milwaukee, WI, USA). A structural MRI scan for subsequent ROI positioning and tissue segmentation was acquired using a 3D fast inversion-recovery prepared gradient echo acquisition (inversion time (TI) = 450 ms, repetition time (TR) = 7 ms, echo time (TE) = 2.8 ms, matrix = 256 × 256 × 124 with a 0.9375 × 0.9375 × 1.1 mm^3^ voxel size). [1H]MRS spectra were acquired using Meshcher–Garwood Point*-*Resolved Spectroscopy (MEGAPRESS). MEGAPRESS is a widely used spectral editing technique that allows the quantification of GABA in humans at magnetic field strengths in which GABA is not readily detectable using plain point-resolved spectroscopy (PRESS) alone^41^. Excitation and refocusing pulses were shifted 2 ppm upfield from water, such that the voxel’s position was correct for metabolites lying in the center of the spectrum, and to minimize chemical shift displacements in the volume selection for the metabolite range of interest. Glutamate and glutamine were estimated from the “unedited” MEGAPRESS spectra (which is equivalent to a PRESS spectrum), as in the “edited” spectra coediting with GABA occurs^42^. GABA was quantified from the “difference” (edited–unedited) spectra. One ROI (35 × 30 × 25 mm) was placed in the left striatum. A second ROI (25 × 40 × 30 mm) was placed on the mPFC. Acquisition parameters were: TR = 2000 ms, TE = 68 ms and a total of 368 averages per ROI: 176 edit-on, 176 edit-off, and 16 with unsuppressed water for water scaling. [1H]MRS spectra were processed using LCModel software (version 6.3-0I; http://s-provencher.com/lcmodel.shtml). Water scaling was performed by LCModel, i.e., each metabolite peak was normalized by expressing its magnitude as a ratio against the magnitude of the unsuppressed water peak. Water-scaled estimates were comparable across ROIs and across participants and are expressed in institutional units as described previously^11^. Spectra were visually inspected, and LCModel quality control parameters were reviewed to verify that spectra were not qualitatively abnormal: all signal-to-noise ratios were ≥14 (striatum: 20.70 ± 3.35; mPFC: 21.10 ± 3.76) and all full widths at half maximums were ≤0.11 ppm (striatum: 0.07 ± 0.015 ppm; mPFC: 0.05 ± 0.022 ppm). Poorly fitted spectra with Cramer–Rao lower bounds equal to or higher than 20% of the metabolic estimate for either glutamate, glutamine or GABA, were excluded from further analysis; this led to the exclusion of one Glx and one GABA estimate from the mPFC ROI of a participant in the control group. The quality of acquired [1H]MRS spectra is shown in Supplementary Figure 1b–d.

To control for interindividual differences in ROI tissue composition, a scaling procedure was used, as previously described^11^. In brief, the structural MRI was first segmented into gray matter (GM), white matter (WM), and cerebrospinal fluid (CSF) using Statistical Parametric Mapping software (SPM2; http://spm.ion.ucl.ac.uk). For each spectroscopy ROI, its position was registered to the segmented structural images and the GM, WM, and CSF content of the ROI was calculated using in-house software. Absolute metabolite values were then calculated from the raw water-scaled LCModel (version 6.3-0I; http://s-provencher.com/lcmodel.shtml) outputs as follows:

$$\begin{array}{ccc}&&{\mathrm {Metab}}_{\mathrm {absolute}} = {\mathrm {Metab}}_{{\mathrm {raw}}}\\ &&\times ({\mathrm {GM}} + {\mathrm {WM}} + \left( {1.55 \times {\mathrm {CSF}}} \right)/{\mathrm {GM}} + {\mathrm {WM}})\end{array}$$

where GM, WM, and CSF together sum up to 1. The (1.55 × CSF) term is included because CSF contains more water than GM or WM, and thus CSF tends to lower water-scaled estimates of the metabolites, in addition to the usual partial volume effects^11^. In the striatum, the groups did not differ on mean tissue contribution of GM, WM, or CSF as indicated by independent-samples unpaired two-tailed *t*-tests (all *p-*values >0.44) (controls: GM 52.5%, WM 43.3%, CSF 8.4%; ASD: GM 52.4%, WM 43.8%, and CSF 8.0%). In the mPFC, there was a significant group difference in GM contribution, which was higher in the ASD group (controls: 57.0%; ASD: 58.7%; *p* = 0.01; two-tailed *t*-test). The GM increase possibly reflects altered cortical neuroanatomy in the ASD group^43^. As brain structure is not the focus of the present study, however, we did not attempt to further characterize this difference but instead entered GM contribution percentage as a covariate in the group comparisons in the mPFC. No differences were seen in WM (controls: 25.8%; ASD: 24.3%; *p* = 0.19; two-tailed *t*-test) or CSF contribution (controls: 20.6%; ASD: 20.3%; *p* = 0.70; two-tailed *t*-test).

### Rodent models

[1H]MRS investigations were carried out in five mouse models and one rat model of ASD. Mouse models included: mice prenatally exposed to VPA and age-matched saline-exposed controls (Harlan Laboratories, Horst, Netherlands; CD1 background), BTBR T+tf/J mice (The Jackson Laboratory, Bar Harbor, ME, USA) and age-matched wild-type C57BL/6J controls (The Jackson Laboratory, Bar Harbor, ME, USA), 15q11-13 patDP mice (kindly provided by T. Takumi, RIKEN, Wako, Japan; C57BL/6J genetic background), *Shank3* KO mice (The Jackson Laboratory, Bar Harbor, ME, USA; C57BL/6J genetic background), *Nlgn3*^R451C^ KI mice (The Rockefeller University, NY, USA; C57BL/6J genetic background), and respective wild-type littermates. In addition, *Nlgn3* KO rats and their wild-type littermates (Horizon Discovery, Boyertown, PA, USA; Sprague-Dawley genetic background) were used. An overview of rodent models and their behavioral characteristics is given in Table 2. A total of 75 ASD-like animals and corresponding 73 controls were investigated in groups of 7–15 animals per condition (Table 2). The targeted sample size of ideally ≥12 animals per condition was based on previous in-house variability assessments^44^ and had to be reduced for logistical reasons in some cases. The animals were selected randomly from different litters. All animals were male adults aged 12–16 weeks (rodent equivalent of the age range of human study-participants)^45^ and weighing, depending on species and strain, 20–50 g (mice) and 350–500 g (rats) at the time of the study. Animals were group-housed (two to three per cage) in a temperature-, humidity-, and light-controlled environment (22–24 °C, 40–60%, 12 h light/dark cycle) and had access to food and water ad libitum. Animal experiments were approved by the Federal Food Safety and Veterinary Office of Switzerland (references BS-2359 and BS-2523) and conducted in strict adherence to the Swiss federal ordinance on animal protection and welfare, as well as according to the rules of the Association for Assessment and Accreditation of Laboratory Animal Care International.

**Table 2:** Summary of etiological and behavioral characteristics of rodent models of ASD

Rodent models and their controls	*n*	Social behavior	Communication	Repetitive behavior	Reference
VPA- vs. saline-treated mice	8 + 7	Impaired	Altered USVs	Increased	^27^
BTBR T+tf/J vs. C57Bl/6J WT mice	15 + 15	Impaired	Altered USVs	Increased	^34^
15q11-13 patDp vs. WT mice	10 + 10	Impaired	Altered USVs	No change	^28– 31^
*Shank3* KO vs. WT mice	15 + 15	Impaired	Altered USVs	Increased	^26, 32^
*Nlgn3*^*R451C*^ KI vs. WT mice	15 + 14	Impaired	Altered USVs	No change	^25^
*Nlgn3* KO vs. WT rats	12 + 12	Impaired	Altered USVs	Increased	^33^

All the mouse models were on a C57BL/6J background, except for VPA (CD1) and BTBR T+tf/J mice. The rat model was on a Sprague-Dawley background. Wild-type littermates were used as controls, except for VPA and BTBR T+tf/J mice for which age-matched saline-treated CD1 and wild-type C57BL/6J mice served as respective controls

*ASD* autism spectrum disorder, *KI* knock-in, *KO* knockout, *patDp* paternal duplication, *USVs* ultrasonic vocalizations, *VPA* valproic acid, *WT* wild type

### Rodent [1H]MRS: data acquisition and processing

[1H]MRS in rodents was performed on a Biospec 9.4T/20 cm horizontal bore small animal MR scanner (Bruker, Ettlingen, Germany), equipped with an actively decoupled two-coil system consisting of a resonator for signal excitation, and a head surface coil for reception. For [1H]MRS, mice were initially anesthetized with 2.5–3% isoflurane (Abbott, Baar, Switzerland) in carrier gas composed of oxygen and air (1:5 v/v) supplied with a face mask to the freely breathing animal. Subsequently, etomidate (Etomidate-®Lipuro; B. Braun Melsungen AG, Melsungen, Germany) was administered as a primed continuous intravenous infusion according to the procedures and conditions reported previously^45^. Rats were anesthetized with isoflurane only: isoflurane concentration was adjusted between 2.0 and 2.3% to maintain stable respiratory rates of 60 breaths/min. Animals were placed in a custom-made cradle and their heads were immobilized in a stereotaxic frame. Respiratory rate, rectal body temperature, and CO_2_ level in the exhaled air were continuously monitored throughout the experiment as previously described^46,47^. Body temperature was maintained at 37 °C using a feedback-regulated electric heating blanket.

Anatomical reference images were obtained by rapid acquisition with refocused echoes (RARE)^48^ (TR = 1800 ms, TEeff = 36 ms, RARE-factor 8, matrix 256 × 256 over 20 × 20 mm and 40 × 40 mm for mice and rats, respectively). Two ROI homologous to those in humans were selected in the right striatum and the mPFC. The right striatum was chosen based on the purported lack of metabolite lateralization and to ensure compatibility with our previous studies^44^ and an extensive in-house database of rodent [1H]MRS data. The ROIs encompassed ~6 µl in mice (striatum: 2 × 1.8 × 1.8 mm; mPFC: 1.5 × 2.6 × 1.5 mm) and ~16 µl in rats (striatum and mPFC: 2.5 × 2.5 × 2.5 mm). Examples of ROI locations are shown in Supplementary Figure 2a, b. Local magnetic field homogeneity was adjusted for both ROIs separately by applying Fastmap^49^. [1H]MRS was carried out by PRESS, as the high magnetic field of 9.4T allows direct detection of GABA without using spectral editing techniques such as MEGAPRESS^50^. Acquisition parameters were: TR = 2000 ms and TE = 10 ms, 4 kHz spectral width sampled with 2048 complex data points and 512 averages collected over 17 min. The PRESS sequence was modified such that the frequencies of the excitation (1.35 ms hermite shape, 4 kHz bandwidth) and refocusing (1.2 ms hermite shape, 2.85 kHz bandwidth) pulses were shifted 2 ppm upfield from water such as to minimize chemical shift displacements in the volume selection for the metabolite range of interest. Water suppression was accomplished using a series of variable power radio frequency pulses on the water resonance^51^, interleaved with outer volume suppression with adiabatic pulses (1 mm gaps to ROI, 5 mm saturation slabs). An unsuppressed water spectrum was obtained immediately preceding each acquisition of a metabolite spectrum using identical acquisition parameters but only eight averages^52^. Quantitative analysis of the [1H]MRS data were carried out using LCModel software (version 6.3-0D) in a fully automated pipeline. The basis set comprised model spectra for 17 metabolites and eight macromolecular/lipid components. Absolute metabolite concentrations were determined with reference to unsuppressed water, uncorrected for relaxation. Spectral fits were reviewed visually to verify that spectra were qualitatively normal. The quality of acquired [1H]MRS spectra is shown in Supplementary Figure 2a,b. Predefined exclusion criteria comprised poor signal-to-noise ratio (five or lower) and head movement detected during data acquisition. Furthermore, glutamine estimates were excluded when they exceeded 3.5 mM (this value representing our empirical in-house threshold separating subjects with abnormally high-glutamine levels^53^ from subjects with normal glutamine levels). Only the latter criterion led to exclusion of data points, namely ten glutamine estimates from mPFC and striatum of five animals.

While there was no blinding with respect to the animals’ genotype/strain during data acquisition, processing, or statistical analysis, the entire pipeline was fully automated, and apart from visual inspection of the spectra there was no step at risk of introducing user bias.

### Statistical analyses

Mean metabolite concentrations were compared pairwise between individuals with ASD and controls using two-tailed independent-samples *t*-tests with *p* < 0.05 as the significance criterion. The same approach was used to compare the mean metabolite concentrations between the animal model groups and their respective control groups. Tests were performed independently for each group, metabolite and ROI, yielding 44 comparisons (humans: eight; animals: 36) for neurometabolites of primary interest (glutamate, glutamine, Glx, and GABA) and 56 comparisons (humans: eight; animals: 48) for metabolites of secondary interest (creatine, choline, *N*-acetylaspartate + *N*-cetylaspartylglutamate (NAA + NAAG), and myo-inositol). To account for these multiple comparisons, the false discovery rate (FDR) was estimated within each of these two priority levels, based on the *p*-value distribution and its deviation from a uniform distribution, as previously described^54,55^. Comparisons between ASD cases or animal model groups and their respective control groups yielded 14 significant differences of neurotransmitters and 11 significant differences of further metabolites of secondary interest. Expected FDR levels were estimated to be 5% and 27%, respectively (i.e., expected numbers of false positives are around 1 and 3). Especially for the metabolites of primary interest, this number is reasonably low, while it seems acceptable for the other metabolites, given that the latter are only meant to provide auxiliary aspects of the disease endophenotype. Residuals per group, metabolite, and ROIs were tested for deviations from a normal distribution and for unequal variances between the conditions compared (i.e., ASD (model) vs. control). None of these tests passed FDR control across conditions, indicating no evidence of violation of *t*-test assumptions.

For the human study, correlations among metabolite concentrations and clinical measures were calculated using Pearson’s product moment coefficient. JMP 10 (SAS Institute, Inc., Cary, NC, USA) and GraphPad Prism 6 (GraphPad Software Inc., La Jolla, CA, USA) software were used for data analysis and preparation of graphs.

## Results

### [1H]MRS in individuals with ASD

**Fig. 1:**
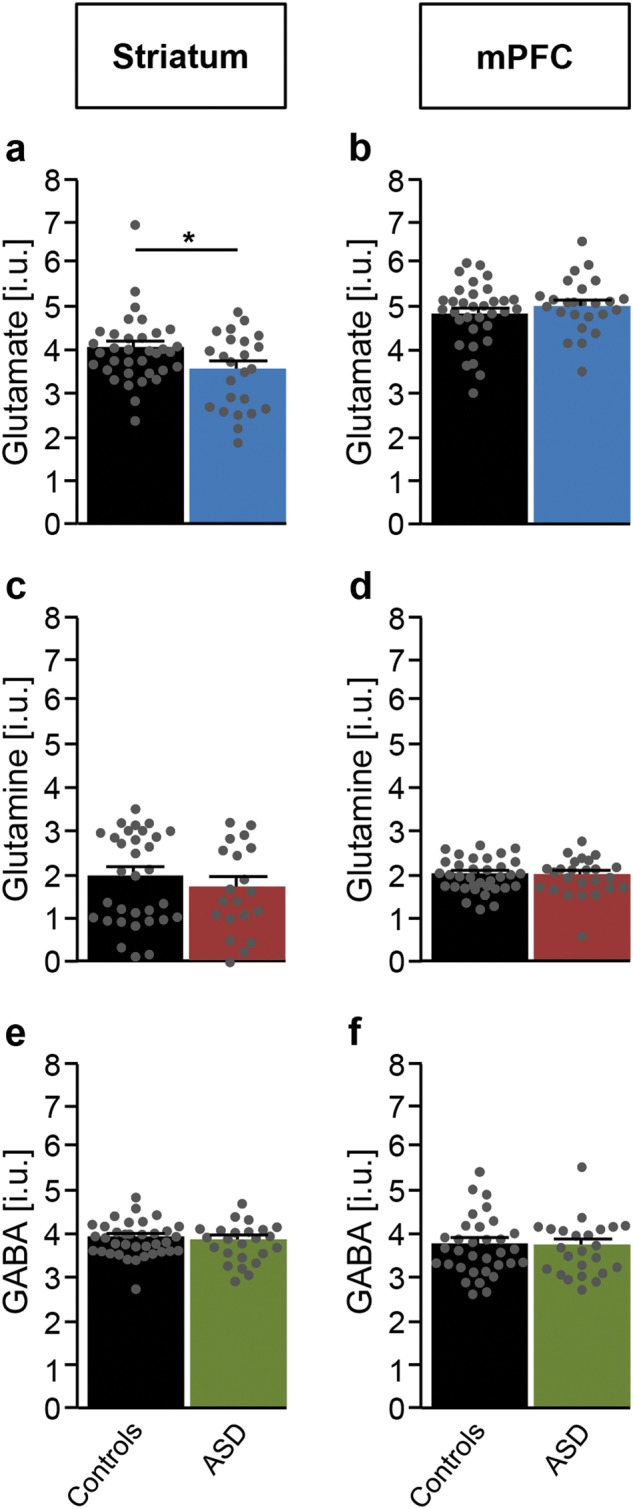
Comparison of glutamate, glutamine and GABA levels observed by [1H]MRS in striatum and mPFC of individuals with ASD and control subjects. Left panels: striatal levels (in institutional units) of **a** glutamate, **c** glutamine, and **e** GABA. Right panels: mPFC levels (in institutional units) of **b** glutamate, **d** glutamine, and **f** GABA. Data are depicted as mean ± SEM of *n* = 25–36 per group; *p < 0.05; two-tailed *t*-test. This difference remains significant with the removal of the putative outlier in the control group, *p* = 0.037. ASD autism spectrum disorder, i.u. institutional units, mPFC medial prefrontal cortex. For additional data see Supplementary Table 1

In this cohort of adult males with idiopathic ASD (Table 1), we observed a significant reduction of glutamate in the striatum as compared with healthy controls (Fig. 1a; Supplementary Table 1). Glx (combined pools of glutamate, glutamine and glutathione) was also decreased (controls: 5.80 ± 0.25; ASD: 4.92 ± 0.35; *p* = 0.04; two-tailed *t*-test; Supplementary Table 1). Yet, no differences were seen in glutamine (Fig. 1c; Supplementary Table 1) or in GABA (Fig. 1e; Supplementary Table 1) in this brain region. In the mPFC, there was no difference in glutamate (Fig. 1b; Supplementary Table 1), glutamine (Fig. 1d; Supplementary Table 1), Glx (controls: 6.77 ± 0.14; ASD: 6.93 ± 0.16; *p* = 0.47; two-tailed *t*-test; Supplementary Table 1), or GABA (Fig. 1f; Supplementary Table 1). For the concomitantly detected cerebral metabolites creatine, NAA + NAAG, and myo-inositol, no differences were observed in either of the two brain regions (Supplementary Table 2). Choline was increased in both striatum and mPFC of people with ASD (Supplementary Table 2).

As striatal glutamate and Glx differed between groups, we examined whether these metabolite levels were correlated with current ASD symptom severity scores as measured using the ADOS, the current “gold standard” clinician-rated measure of core ASD behaviors. Striatal glutamate and Glx were both significantly, negatively correlated with ADOS social impairments: ASD individuals with lower (more abnormal) glutamate and Glx displayed worse social function (glutamate: *r* = −0.56, *p* = 0.005; Glx: *r* = −0.63, *p* = 0.002; both significant after Bonferroni correction for two ROIs × two ADOS scales). There was no correlation between striatal glutamate or Glx and the severity of repetitive behaviors (glutamate: *r* = −0.07, *p* = 0.77; Glx: *r* = −0.07, *p* = 0.77).

### [1H]MRS in rodent models of ASD

In parallel to the human [1H]MRS studies, we evaluated the neurochemical differences in six rodent models of ASD with different underlying etiologies that emulate the breadth of potential risk factors in idiopathic ASD (Table 2). Across the models several neurochemical differences were observed.

**Fig. 2:**
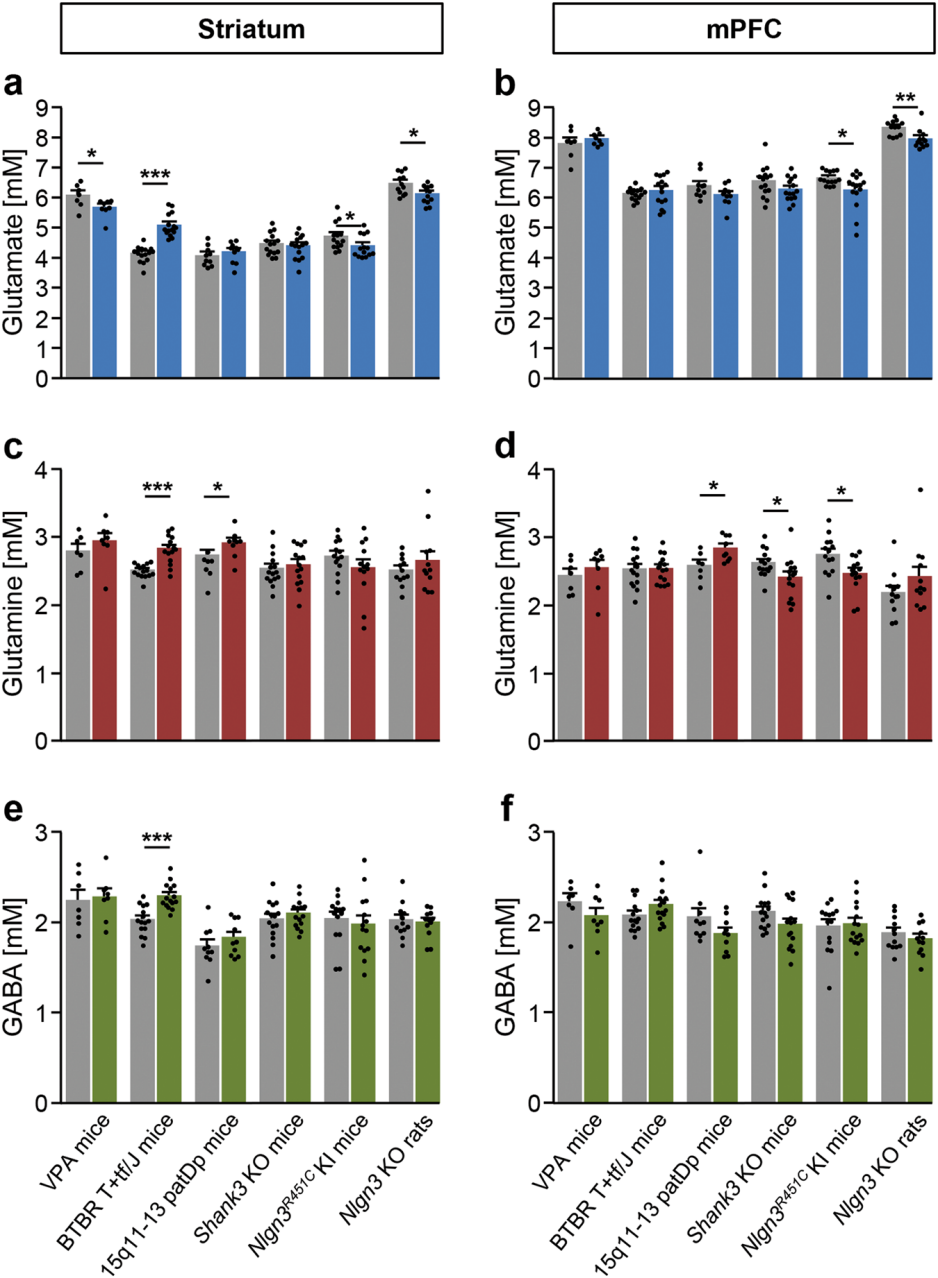
Comparison of glutamate, glutamine and GABA concentrations observed by [1H]MRS in the striatum and mPFC of rodent models of ASD and their corresponding controls. Left panels: concentrations (in mM) of **a** glutamate, **c** glutamine, and **e** GABA in striatum. Right panels: concentrations (in mM) of **b** glutamate, **d** glutamine, and **f** GABA in mPFC. All the mouse models (colored bars) were on a C57BL/6J background, except for VPA (CD1) and BTBR T+tf/J mice. The rat model (colored bars) was on a Sprague-Dawley background. Wild-type (gray bars) littermates were used as controls, except for VPA and BTBR T+tf/J mice for which age-matched saline-treated CD1 and wild-type C57BL/6J mice served as respective controls. Note the strain-dependent differences in glutamate levels. Data are depicted as mean ± SEM of *n* = 7–15 per group; *p < 0.05, **p < 0.01, ***p < 0.001; two-tailed *t*-test. KI knock-in, KO knockout, mPFC medial prefrontal cortex, patDp paternal duplication, VPA valproic acid. For additional data see Supplementary Table 2

A decrease in striatal glutamate was the most consistent finding, being observed in *Nlgn3*^*R451C*^ KI mice, *Nlgn3* KO rats, and in mice prenatally exposed to VPA (Fig. 2a; Supplementary Table 3). In *Nlgn3*^*R451C*^ KI mice and *Nlgn3* KO rats, a decrease in glutamate levels was also seen in the mPFC (Fig. 2b; Supplementary Table 3).

Among the other models, different patterns of changes were seen. For instance, glutamate and glutamine were elevated in the striatum of BTBR T+tf/J mice (Fig. 2a, c; Supplementary Table 3). Glutamine was elevated in the striatum and mPFC of 15q11-13 paternal duplication (patDp/+; henceforth referred to as 15q11-13 patDp) mice, whereas reduced glutamine levels were observed in the mPFC of *Nlgn3*^*R451C*^ KI and *Shank3* KO mice (Fig. 2d; Supplementary Table 3). Our observation of no alterations in GABA in humans with ASD was recapitulated in most of the rodent models, including *Nlgn3*^*R451*^ KI mice, *Nlgn3* KO rats, and VPA mice (Fig. 2e, f; Supplementary Table 3). Elevated GABA levels were observed only in the striatum of BTBR T+tf/J mice (Fig. 2e; Supplementary Table 3).

Several brain region- and model-specific changes were also noted in other commonly reported neurometabolites (Supplementary Table 4). Myo-inositol levels were altered in the striatum and mPFC of *Nlgn3* KO rats and BTBR T+tf/J mice (Supplementary Table 4). In BTBR T+tf/J mice, creatine was increased in the striatum but strongly decreased in the mPFC (Supplementary Table 4). In contrast, in *Nlgn3* KO rats creatine was elevated in the mPFC (Supplementary Table 4). Levels of NAA + NAAG and choline-containing metabolites were only altered in BTBR T+tf/J mice (Supplementary Table 4).

## Discussion

In this translational [1H]MRS study, we examined glutamate and GABA levels in people with ASD and in six rodent models of the disorder. Consistent with the concept of a glutamate/GABA imbalance in ASD, we found that adults with idiopathic ASD have decreased glutamate concentration in the striatum compared to controls. The association between striatal glutamate and the severity of social impairment implies that this abnormality is clinically significant. In contrast to our findings with glutamate, we observed no alterations in GABA in adults with ASD. Further, we showed that reduced striatal glutamate is recapitulated in a subset of rodent models, namely in mice exposed prenatally to valproate, and in both mice and rats carrying mutations in the ASD-associated gene *Nlgn3* although in these models mPFC glutamate was also reduced.

Our current human [1H]MRS results replicate and extend our previous findings of reduced Glx in the striatum, but not in the cortex, of people with ASD^11^. We are now also able to confirm that it is the excitatory neurotransmitter glutamate that is reduced in the striatum in ASD. This is because of the higher magnetic field strength of the 3T MRI scanner used in the present study vs. 1.5T in our previous work^11^, which allows for the separate quantification of glutamate and glutamine, and minor contributions of glutathione. The glutamate reduction is regionally specific, however, which may explain the differing findings of cortical glutamate across various studies in which [1H]MRS was performed in different cortical areas^8–16^. Brain region- and even neural circuit-specific changes in the E/I balance have also been observed in animal models of ASD^6,22–26^.

In the present study, we did not observe differences in GABA concentration in adults with ASD in either the striatum or in the mPFC. However, reductions in GABA have been observed in [1H]MRS studies of children with ASD^13,17–20^. Therefore, GABA abnormalities in ASD could be age-dependent. Future research should examine GABA longitudinally over the life course—both in humans and in rodent ASD models—in order to determine whether the maturational trajectory of the GABA system is altered in ASD.

We assessed six rodent models of ASD as part of this translational study, and showed that the abnormality seen in individuals with ASD—namely a striatal glutamate deficit—was recapitulated in VPA-exposed mice, *Nlgn3*^*R451C*^ KI mice, and *Nlgn3* KO rats. Our novel finding is that these rodent models of ASD are similar to each other with regard to this key neurochemical change, and that they recapitulate the change seen in human idiopathic ASD, thus pointing to a common pathophysiology. Literature on valproate and on *Nlgn3* offers intriguing clues that support this notion of a common pathophysiology. Valproate is an anticonvulsant and antimanic agent with multiple direct pharmacological effects, such as enhancing GABA neurotransmission^56^. VPA is also known to modulate gene expression via inhibition of histone deacetylase^56^, and in particular, VPA has been shown to reduce expression of NLGN3^57^, thus mimicking the effect of the genetic manipulation in *Nlgn3*^*R451C*^ KI mice and *Nlgn3* KO rats. Both point mutations^58^ and deletions^59,60^ of this gene have been identified in people with ASD, and are modeled by *Nlgn3*^*R451C*^ KI mice and *Nlgn3* KO rats, respectively.

The mechanism by which mutations in the postsynaptic cell-adhesion protein NLGN3 affects the striatal glutamate system is unclear. Nevertheless, its involvement in synapse function and the clustering of synaptic vesicles in the presynaptic buttons^25,26,61,62^ suggests that NLGN3 deficiency may result in a decreased number of glutamate-containing presynaptic vesicles. Unlike in humans with ASD, *Nlgn3* mutant mice and rats also showed reduced glutamate in the mPFC, suggesting that the abnormalities were less regionally specific perhaps due to the pervasiveness of the genetic alterations in the brains of these models.

We also observed a tendency toward reduction in striatal glutamate and glutamine levels in the *Shank3* KO mice. SHANK3, like NLGN3, is a synaptic protein implicated in ASD pathology^26,63^, and it also signals through the neuroligin–neurexin complex^64^. This implies both a pathophysiological convergence in SHANK3- and NLGN3-deficient rodents and a potential mechanism underpinning the regionally specific neurochemical abnormalities that we observed in humans.

Our finding of altered striatal glutamate might appear surprising, given that the striatum is primarily composed of GABAergic medium spiny neurons in which glutamate levels are very low^65,66^. However, glutamate is contained within glutamatergic inputs from the cerebral cortex, i.e., corticostriatal projections, in this region. Hence, the [1H]MRS-observable reduction in striatal glutamate in both people with ASD, and in particular animal models of the disorder, might reflect the presence of fewer (or less effective) corticostriatal projections. This interpretation is in line with reports that individuals with ASD have impaired long range connectivity^67^ and reduced neural activity in corticostriatal circuits in response to a variety of social rewards^68,69^. Thus our finding of a correlation between striatal glutamate levels and the severity of social deficits is consistent with the role of striatum in social behaviors^36,37^.

We observed reduced striatal glutamate in humans with ASD and more prevalently in several ASD rodent models. Based on observations that the MRS-amenable glutamate and GABA pools relate to neural function^44^, this result is opposing the original hypothesis of neural excitability in ASD put forward by Rubenstein and Merzenich^5^. This hypothesis of increased excitation was largely based on the observation that epilepsy frequently co-occurs with ASD^5^. However, the majority of ASD individuals do not have seizures^70^, and epilepsy is not simply a consequence of an increase in neuronal excitation^71^. Moreover, work on other animal models of ASD supports the idea of a shift in the E/I balance away from excitation^24^.

Our study has some limitations. While [1H]MRS offers the unique possibility of noninvasively measuring metabolite concentration across species, a restriction of the technique is that it measures the total concentration of metabolites, e.g., glutamate, within a selected ROI and cannot distinguish between synaptic, neuronal and glial pools. However, for glutamate, there is indication that the neurotransmitter and metabolic pools are correlated^44,72^, and glial and extracellular glutamate pools are several-folds smaller than the neuronal pool^65,73,74^. Therefore, glutamate concentration measured by [1H]MRS can be viewed as proportional to the neuronal neurotransmitter pool.

Regarding the limitations of the human [1H]MRS study, our sample size was modest. However, it was comparable with, or larger than, similar studies^8–20^ and our key finding of reduced striatal Glx in ASD replicates our previous study in a new cohort of participants^11^. We excluded subjects with a below-normal IQ or a history of epilepsy or seizures. This was done in order to ensure that our sample was broadly representative of the ASD population, since the majority of ASD cases do not have epilepsy^70^ and recent studies suggest that most have normal range IQ^75^. Nonetheless, many individuals with ASD do exhibit these features and therefore more research would be required to confirm whether our results hold in such cases. A further limitation is that we were unable to estimate true absolute concentrations in the human [1H]MRS. The estimates we calculated are comparable between individuals, but cannot be directly interpreted as concentrations (in mM) because of a number of assumptions made in the analysis of [1H]MRS data in LCModel. For instance metabolite estimates vary depending on how macromolecules are estimated and the method we used (estimating eight macromolecule peaks) has been scrutinized^51^. This, however, does not affect the validity of our between-group comparisons because the same analysis was used for all spectra.

A further, conceptual limitation is that our animal models (except BTBR T+tf/J mice) are based on specific etiological factors, and do not model idiopathic ASD as such. However, there are no animal models of idiopathic ASD in this sense, as even BTBR T+tf/J mice were reported to share some genetic features with clinical ASD^76–78^. Our hypothesis was that individuals with ASD and rodent models of ASD may share key pathophysiological mechanisms regardless of etiology^79^. In order to identify possible points of convergence that would guide future mechanistic investigations, we selected a range of animal models, while restricting our selection to “non-syndromic” ASD models as syndromic forms of ASD, associated with gross neuroanatomical, and morphological abnormalities, could represent unusual subtypes.

In the rodent study, our use of anesthesia to facilitate in vivo [1H]MRS constitutes a difference from our human studies, but the alternative of scanning awake animals, would also have introduced bias due to stress experienced by the rodents^80^. We have previously demonstrated reliability of [1H]MRS in detecting changes in glutamate and GABA in anesthetized animals^44^. We cannot completely exclude interactions of anesthesia with neurotransmitter levels, e.g., elicited by the anesthetics’ effect on GABA_A_ receptors^81^. However, as we used the same anesthetic regime for all the animal models and their controls, it is unlikely that this could explain the differences in glutamate and GABA between rodent groups. Cross-species matching by age (or developmental and maturational stage) for a direct comparison of human and rodent data is complex. Notwithstanding, mice aged 3–4 months, as used in this study, have been reported to reflect a human equivalent of early adulthood^45^, thus broadly reflecting the human cohorts. Also, we did not perform [1H]MRS and behavioral assessments in the same animals to avoid potential confounds related to the series and sequence of experimental procedures. Previous work has shown that behavioral testing could induce changes in neurometabolite levels^82^. On the other hand, the imaging procedure with 1H of anesthesia could impact upon subsequent behavioral testing.

As practical considerations limited us to a single unilateral striatal ROI, we chose the left striatum in humans and the right striatum in our animal models in order to maintain maximum compatibility with our former studies^11,44^ and extensive in-house databases. Earlier studies^38–40^, as well as our separate [1H]MRS study performed in the left striatum of saline-treated C57BL/6J and BTBR T+tf/J mice reported levels for glutamate (C57BL/6J: 4.88 ± 0.08; BTBR T+tf/J: 5.61 ± 0.11), glutamine (C57BL/6J: 2.52 ± 0.08; BTBR T+tf/J: 2.96 ± 0.08), and GABA (C57BL/6J: 2.08 ± 0.05; BTBR T+tf/J: 2.42 ± 0.06) that closely mirrored those measured here in the right striatum of those two mouse strains, thus suggesting the absence of metabolite lateralization.

Finally, another technical difference between our human and animal studies was the difference in [1H]MRS magnetic field strength and pulse sequence used (3T MEGAPRESS vs. 9.4T PRESS) along with ROI size differences. However, both of these are methods optimized and qualified for the two species, and there is no a priori reason to expect them to differ in their ratiometric measurements.

In conclusion, in this first translational [1H]MRS study in ASD, we replicated and extended our previous finding that high-functioning adults with idiopathic ASD have reduced glutamate in the striatum^11^, while we detected no change in GABA. We further showed that the reduction in striatal glutamate is related to core ASD symptom severity. Moreover, reduction in striatal glutamate was recapitulated in rodents carrying mutations in *Nlgn3* or being prenatally exposed to VPA. These translational findings support the notion of glutamatergic dysfunction in the corticostriatal pathway as an underlying core pathophysiological mechanism of ASD. However, contrary to the original hypothesis put forward by Rubenstein and Merzenich^5^ that ASD might be rooted in increased cortical excitability, our data on humans, as well as ours and recent work^24^ on several rodent models of ASD, are suggestive of a region-specific imbalance slanted toward reduced excitation.

## Electronic supplementary material

Supplemental Material
