# Supplementary material for: Glutamate and GABA in autism spectrum disorder—a translational magnetic resonance spectroscopy study in man and rodent models
Source: Transl Psychiatry. 2018 May 25;8:106. doi: 10.1038/s41398-018-0155-1 (PMC5970172; doi:10.1038/s41398-018-0155-1)
Supplement: Supplementary file 1 — Supplemental Material [file 41398_2018_155_MOESM1_ESM.docx]

Glutamate and GABA in autism spectrum disorder – A translational magnetic resonance spectroscopy study in man and rodent models

Jamie Horder, PhD^1,7^, Marija M. Petrinovic, PhD^2,7,9^, Maria A. Mendez, PhD^1^, Andreas Bruns, PhD^2^, Toru Takumi, PhD^3^, Will Spooren, PhD^2^, Gareth J. Barker, PhD^4^, Basil Künnecke, PhD^2,8^, Declan G. Murphy, MD^1,5,6,8^

^1^Department of Forensic and Neurodevelopmental Sciences, Institute of Psychiatry, Psychology and Neuroscience, King’s College London, De Crespigny Park, London SE5 8AF, UK.

^2^Roche Pharma Research & Early Development, Neuroscience, Roche Innovation Center Basel, F. Hoffmann-La Roche Ltd, Grenzacherstrasse 124, CH-4070 Basel, Switzerland.

^3^RIKEN Brain Science Institute, Wako, Japan.

^4^Centre for Neuroimaging Sciences, Institute of Psychiatry, Psychology and Neuroscience, King’s College London, De Crespigny Park, London SE5 8AF, UK.

^5^Autism Assessment and Behavioural Genetics Clinic, South London and Maudsley NHS Foundation Trust, Bethlem Royal Hospital, Beckenham, UK.

^6^Sackler Institute for Translational Neurodevelopment, Institute of Psychiatry, Psychology and Neuroscience, King’s College London, London, UK.

^7^Shared first authorship. These authors contributed equally to this work.

^8^Shared last authorship. These authors contributed equally to this work.

^9^Present address: Department of Forensic and Neurodevelopmental Sciences, Institute of Psychiatry, Psychology and Neuroscience, King’s College London, De Crespigny Park, London SE5 8AF, UK, and Sackler Institute for Translational Neurodevelopment, Institute of Psychiatry, Psychology and Neuroscience, King’s College London, London, UK.

***Corresponding author:** Marija M. Petrinovic, Department of Forensic and Neurodevelopmental Sciences, and the Sackler Institute for Translational Neurodevelopment, Institute of Psychiatry, Psychology and Neuroscience, King’s College London, 16 De Crespigny Park, London SE5 8AF, UK

Email: [marija-magdalena.petrinovic@kcl.ac.uk](mailto:marija-magdalena.petrinovic@kcl.ac.uk); Phone: +44 207 848 0550; Fax: +44 207 848 0650

**
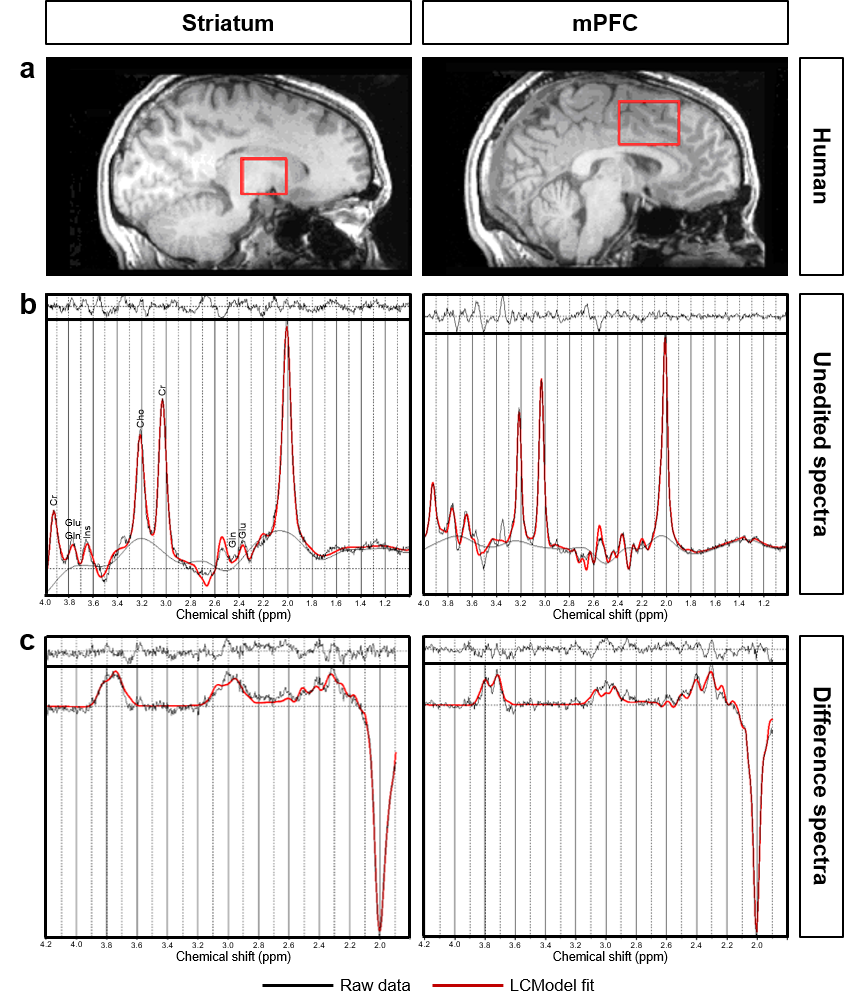
**

**Figure 1.** **Examples of the placement of [1H]MRS region-of-interests and corresponding representative [1H]MRS spectra acquired in humans**. (**a**) The first region of interest (ROI; 35 mm x 30 mm x 25 mm) was selected in the striatum, and included the head of the caudate, the anterior putamen, and the internal capsule. The second ROI (25 mm x 40 mm x 30 mm) was placed on the midline covering the medial prefrontal cortex (mPFC) bilaterally. (**b**, **c**) Representative *in vivo* [1H]MRS (**b**) unedited, and (**c**) difference (edited - unedited) spectra from control subjects are shown for each ROI with the raw spectrum (black line) and superimposed LCModel fit (red line; resulting residuals are shown above the spectra). Spectra were acquired using the MEGAPRESS sequence at 3T.


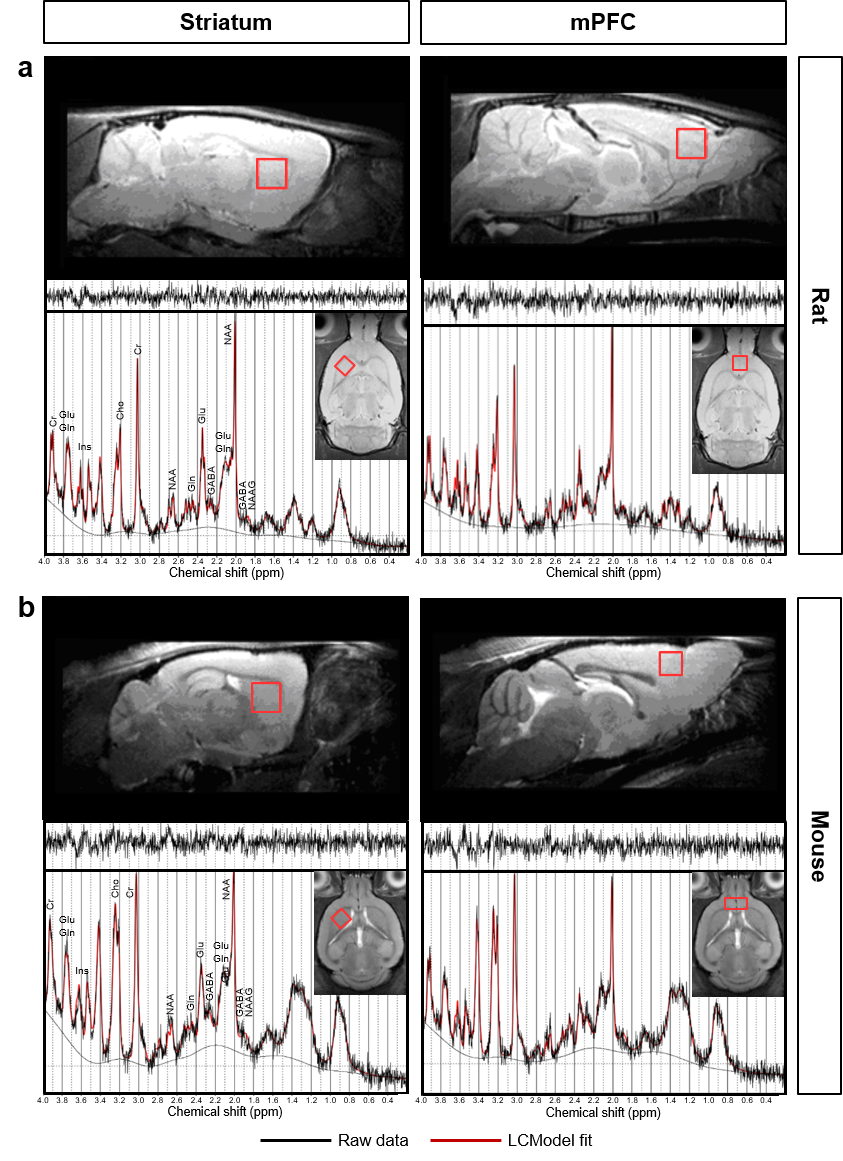


**Figure 2.** **Examples of the placement of [1H]MRS region-of-interests and corresponding representative [1H]MRS spectra acquired in animal models of ASD**. (**a**) **Rats**: ROIs were placed in striatum (2.5 mm x 2.5 mm x 2.5 mm) and mPFC (2.5 mm x 2.5 mm x 2.5 mm). Representative *in vivo* [1H]MRS spectra from wild-type rats with the raw (black line) and superimposed LCModel fit (red line; resulting residuals are shown abow the spectra) are shown for each ROI. Spectra were acquired using the PRESS sequence at 9.4T. (**b**) **Mice**: ROIs were placed in striatum (2 mm x 1.8 mm x 1.8 mm) and mPFC (1.5 mm x 2.6 mm x 1.5 mm). Representative *in vivo* [1H]MRS spectra from wild-type mice with the raw (black line) and superimposed LCModel fit (red line; resulting residuals are shown abow the spectra) are shown for each ROI. Spectra were acquired using the PRESS sequence at 9.4T. Cho, choline; Cr, creatine; Gln, glutamine; Glu, glutamate; Ins, myo-inositol; NAA, N-acetylaspartate; NAAG, N-acetylaspartylglutamate.

**Table 1. Levels of glutamate, glutamine, Glx and GABA determined by [1H]MRS in the striatum and mPFC of individuals with ASD and control subjects**

| **Metabolite** | **Controls** (mean±SEM) | **ASD**  (mean±SEM) | ***p* value** |
| --- | --- | --- | --- |
| **Metabolite levels in striatum [i.u.]** | | | |
| Glutamate | 4.02 ± 0.13  (3.93±0.10)^a^ | 3.52 ± 0.18  (3.52±0.18)^1^ | **0.025**  **0.037**^1^ |
| Glutamine | 1.94 ± 0.19 | 1.68 ± 0.23 | 0.39 |
| Glx | 5.80 ± 0.25 | 4.92 ± 0.35 | **0.04** |
| GABA | 3.90 ± 0.07 | 3.84 ± 0.09 | 0.58 |
| **Metabolite levels in mPFC [i.u.]** | | | |
| Glutamate | 4.78 ± 0.12 | 4.99 ± 0.13 | 0.33 |
| Glutamine | 1.98 ± 0.06 | 1.97 ± 0.09 | 0.85 |
| Glx | 6.77 ± 014 | 6.93 ± 016 | 0.47 |
| GABA | 3.74 ± 0.12 | 3.71 ± 0.13 | 0.87 |

Data are depicted as mean ± SEM of *n* = 25–36 per group; two-tailed t-test. ^1^statistical analysis following exclusion of a control subject with the highest level of glutamate. ASD, autism spectrum disorder; GABA, gamma-aminobutyric acid; i.u., institutional units; Glx, combined pools of glutamate, glutamine and glutathione; mPFC, medial prefrontal cortex.

**Table 2. Levels of selected neurometabolites determined by [1H]MRS in the striatum and mPFC of individuals with ASD and control subjects**

| **Metabolite** | **Controls** (mean±SEM) | **ASD**  (mean±SEM) | **T value** | **df** | ***p* value** |
| --- | --- | --- | --- | --- | --- |
| **Metabolite levels in striatum [i.u.]** | | | | | |
| Creatine | 8.51 ± 0.14 | 8.63 ± 0.14 | 0.58 | 57 | 0.56 |
| Choline | 1.97 ± 0.04 | 2.12 ± 0.06 | 2.04 | 57 | **0.046** |
| NAA+NAAG | 10.88 ± 0.18 | 10.61 ± 0.21 | 0.96 | 57 | 0.34 |
| Myo-inositol | 4.10 ± 0.11 | 4.14 ± 0.18 | 0.20 | 57 | 0.84 |
| **Metabolite levels in mPFC [i.u.]** | | | | | |
| Creatine | 8.13 ± 0.14 | 8.50 ± 0.10 | 1.92 | 57 | 0.06 |
| Choline | 1.91 ± 0.04 | 2.04 ± 0.04 | 2.27 | 57 | **0.027** |
| NAA+NAAG | 9.70 ± 0.21 | 9.99 ± 0.19 | 0.95 | 57 | 0.35 |
| Myo-inositol | 4.89 ± 0.14 | 5.31 ± 0.15 | 1.93 | 57 | 0.06 |

Data are depicted as mean ± SEM of *n* = 25–36 per group two-tailed t-test. ASD, autism spectrum disorder; df, degrees of freedom; i.u., institutional units; mPFC, medial prefrontal cortex; NAA, N-acetylaspartate; NAAG, N-acetylaspartylglutamate.

**Table 3. Concentrations of glutamate, glutamine and GABA determined by [1H]MRS in striatum and mPFC of rodent models of ASD and their corresponding controls**

| **Metabolite** | |  | **VPA vs.**  **control mice**  (mean±SEM)  (*p* value) | **BTBR vs. C57BL/6J mice**  (mean±SEM)  (*p* value) | **15q11-13 patDp vs.**  **WT mice** (mean±SEM)  (*p* value) | ***Shank3* KO vs. WT mice** (mean±SEM)  (*p* value) | ***Nlgn3^R451C^* KI vs.**  **WT mice** (mean±SEM)  (*p* value) | ***Nlgn3* KO**  **vs. WT rats**  (mean±SEM)  (*p* value) |
| --- | --- | --- | --- | --- | --- | --- | --- | --- |
|  | **Metabolite concentrations in striatum [mM]** | | | | | | | |
| Glutamate | |  | 5.72 ± 0.11  6.12 ± 0.15  *p* = **0.046** | 5.13 ± 0.10  4.19 ± 0.07  *p* < **0.0001** | 4.25 ± 0.11  4.12 ± 0.10  *p* = 0.40 | 4.44 ± 0.10  4.51 ± 0.09  *p* = 0.59 | 4.44 ± 0.10  4.76 ± 0.12  *p* = **0.044** | 6.17 ± 0.09  6.52 ± 0.10  *p* = **0.015** |
| Glutamine | |  | 2.97 ± 0.11  2.82 ± 0.10  *p* = 0.34 | 2.85 ± 0.05  2.54 ± 0.03  *p* < **0.0001** | 2.94 ± 0.07  2.60 ± 0.07  *p* = **0.0038** | 2.62 ± 0.07  2.56 ± 0.06  *p* = 0.56 | 2.57 ± 0.12  2.75 ± 0.07  *p* = 0.20 | 2.68 ± 0.13  2.54 ± 0.06  *p* = 0.33 |
| GABA | |  | 2.29 ± 0.09  2.25 ± 0.11  *p* = 0.78 | 2.31 ± 0.04  2.04 ± 0.04  *p* = < **0.0001** | 1.84 ± 0.06  1.75 ± 0.07  *p* = 0.33 | 2.11 ± 0.04  2.05 ± 0.05  *p* = 0.35 | 1.99 ± 0.09  2.05 ± 0.07  *p* = 0.62 | 2.01 ± 0.05  2.04 ± 0.05  *p* = 0.70 |
|  | **Metabolite concentrations in mPFC [mM]** | | | | | | | |
| Glutamate | |  | 8.00 ± 0.07  7.84 ± 0.18  *p* = 0.40 | 6.27 ± 0.12  6.17 ± 0.05  *p* = 0.45 | 6.14 ± 0.11  6.43 ± 0.12  *p* = 0.09 | 6.32 ± 0.10  6.59 ± 0.13  *p* = 0.12 | 6.30 ± 0.16  6.70 ± 0.06  *p* = **0.027** | 7.99 ± 0.09  8.37 ± 0.07  *p* = **0.004** |
| Glutamine | |  | 2.57 ± 0.11  2.46 ± 0.09  *p* = 0.45 | 2.56 ± 0.05  2.56± 0.07  *p* = 0.96 | 2.86 ± 0.06  2.61 ± 0.08  *p* = **0.018** | 2.43 ± 0.08  2.64 ± 0.05  *p* = **0.027** | 2.49 ± 0.07  2.77 ± 0.08  *p* = **0.013** | 2.44 ± 0.14  2.21 ± 0.09  *p* = 0.18 |
| GABA | |  | 2.09 ± 0.08  2.24 ± 0.09  *p* = 0.24 | 2.21 ± 0.05  2.09 ± 0.04  *p* = 0.09 | 1.89 ± 0.06  2.07 ± 0.09  *p* = 0.10 | 1.99 ± 0.06  2.13 ± 0.05  *p* = 0.08 | 1.99 ± 0.06  1.97 ± 0.07  *p* = 0.78 | 1.83 ± 0.05  1.89 ± 0.05  *p* = 0.37 |

All the mouse models were on a C57BL/6J background, except for VPA (CD1) and BTBR T+tf/J mice. The rat model was on a Sprague-Dawley background. Wild-type littermates were used as controls, except for VPA and BTBR T+tf/J mice for which age-matched saline-treated CD1 and wild-type C57BL/6J mice served as respective controls. Data are depicted as mean ± SEM of *n* = 7–15 per group; two-tailed t-test. ASD, autism spectrum disorder; BTBR, BTBR T+tf/J; GABA, gamma-aminobutyric acid; KI, knock-in; KO, knock-out; mPFC, medial prefrontal cortex; patDp, paternal duplication; VPA, valproic acid; WT, wild-type.

**Table 4. Concentrations of selected neurometabolites determined by [1H]MRS in striatum and mPFC of rodent models of ASD and their corresponding controls**

| **Metabolite** | **VPA vs.**  **control mice**  (mean±SEM)  (*p* value) | **BTBR vs. C57BL/6J mice**  (mean±SEM)  (*p* value) | **15q11-13 patDp vs.**  **WT mice** (mean±SEM)  (*p* value) | ***Shank3* KO vs. WT mice** (mean±SEM)  (*p* value) | ***Nlgn3^R451C^* KI vs.**  **WT mice** (mean±SEM)  (*p* value) | ***Nlgn3* KO**  **vs. WT rats**  (mean±SEM)  (*p* value) |
| --- | --- | --- | --- | --- | --- | --- |
| **Metabolite concentrations in striatum [mM]** | | | | | | |
| Creatine | 2.15 ± 0.12  2.27 ± 0.07  *p* = 0.40 | 2.07 ± 0.14  1.75 ± 0.07  *p* = **0.047** | 1.91 ± 0.09  1.77 ± 0.07  *p* = 0.22 | 1.95 ± 0.06  2.01 ± 0.08  *p* = 0.62 | 2.05 ± 0.08  2.10 ± 0.10  *p* = 0.69 | 2.04 ± 0.08  1.96 ± 0.06  *p* = 0.43 |
| Choline | 0.85 ± 0.06  0.80 ± 0.05  *p* = 0.57 | 0.93 ± 0.04  0.77 ± 0.03  *p* = **0.002** | 0.87 ± 0.05  0.90 ± 0.03  *p* = 0.58 | 0.90 ± 0.04  0.94 ± 0.05  *p* = 0.56 | 1.12 ± 0.06  1.01 ± 0.07  *p* = 0.22 | 0.93 ± 0.03  0.97 ± 0.03  *p* = 0.45 |
| NAA+NAAG | 4.68 ± 0.11  4.95 ± 0.05  *p* = 0.06 | 4.18 ± 0.04  4.15 ± 0.05  *p* = 0.63 | 4.09 ± 0.07  4.07 ± 0.05  *p* = 0.85 | 4.05 ± 0.07  4.05 ± 0.07  *p* = 1.00 | 4.21 ± 0.09  4.29 ± 0.12  *p* = 0.57 | 5.18 ± 0.05  5.14 ± 0.07  *p* = 0.69 |
| Myo-inositol | 4.01 ± 0.08  4.03 ± 0.23  *p* = 0.95 | 3.20 ± 0.07  2.97 ± 0.07  *p* = **0.037** | 2.83 ± 0.08  2.63 ± 0.06  *p* = 0.45 | 3.46 ± 0.08  3.35 ± 0.07  *p* = 0.29 | 3.07 ± 0.11  3.08 ± 0.09  *p* = 0.95 | 3.49 ± 0.08  3.22 ± 0.06  *p* = **0.012** |
| **Metabolite concentrations in mPFC [mM]** | | | | | | |
| Creatine | 1.86 ± 0.08  1.67 ± 0.10  *p* = 0.17 | 1.20 ± 0.76  1.58 ± 0.59  *p* = **0.0005** | 1.63 ± 0.17  1.66 ± 0.08  *p* = 0.85 | 1.49 ± 0.08  1.65 ± 0.06  *p* = 0.12 | 1.90 ± 0.18  1.85 ± 0.10  *p* = 0.83 | 1.96 ± 0.05  1.72 ± 0.06  *p* = **0.009** |
| Choline | 0.70 ± 0.05  0.71 ± 0.04  *p* = 0.95 | 0.81 ± 0.05  0.81 ± 0.04  *p* = 1.00 | 0.90 ± 0.05  0.85 ± 0.04  *p* = 0.52 | 0.81 ± 0.04  0.86 ± 0.04  *p* = 0.45 | 0.95 ± 0.06  0.89 ± 0.05  *p* = 0.46 | 0.82 ± 0.02  0.84 ± 0.03  *p* = 0.76 |
| NAA+NAAG | 5.56 ± 0.13  5.57 ± 0.15  *p* = 0.97 | 5.27 ± 0.06  4.93 ± 0.05  *p* = **0.0001** | 5.10 ± 0.07  5.37 ± 0.11  *p* = 0.13 | 5.15 ± 0.06  5.16 ± 0.04  *p* = 0.93 | 5.32 ± 0.06  5.38 ± 0.06  *p* = 0.52 | 5.95 ± 0.10  6.06 ± 0.13  *p* = 0.42 |
| Myo-inositol | 4.77 ± 0.13  4.69 ± 0.14  *p* = 0.68 | 3.75 ± 0.07  4.44 ± 0.06  *p* < **0.0001** | 4.04 ± 0.14  3.98 ± 0.14  *p* = 0.78 | 4.44 ± 0.08  4.54 ± 0.09  *p* = 0.41 | 3.97 ± 0.14  4.26 ± 0.10  *p* = 0.11 | 4.63 ± 0.07  4.12 ± 0.09  *p* = **0.0002** |

All the mouse models were on a C57BL/6J background, except for VPA (CD1) and BTBR T+tf/J mice. The rat model was on a Sprague-Dawley background. Wild-type littermates were used as controls, except for VPA and BTBR T+tf/J mice for which age-matched saline-treated CD1 and wild-type C57BL/6J mice served as respective controls. Data are depicted as mean ± SEM of *n* = 7–15 per group; two-tailed t-test. ASD, autism spectrum disorder; BTBR, BTBR T+tf/J; KI, knock-in; KO, knock-out; mPFC, medial prefrontal cortex; NAA, N-acetylaspartate; NAAG, N-acetylaspartylglutamate; patDp, paternal duplication; VPA, valproic acid; WT, wild-type.
